# The complete chloroplast genome and phylogenetic analysis of *Astragalus sinicus* Linne 1767

**DOI:** 10.1080/23802359.2022.2074804

**Published:** 2022-05-12

**Authors:** Qinglin Ke, Hongbin Shangguan, Wenqiang Liu, Minqiang Tang, Jianxin Bian, Licao Cui, Yihan Li

**Affiliations:** a College of Bioscience and Engineering, Jiangxi Agricultural University, Nanchang, China; b College of Forestry, Hainan University, Haikou, China; c Institute of Advanced Agricultural Sciences, Peking University, Weifang, China

**Keywords:** Chloroplast genome, phylogenetic analysis, *Astragalus sinicus*

## Abstract

*Astragalus sinicus* Linne 1767 is a traditional winter-growing green manure, that plays an important role in upgrading soil fertility and maintaining crop yield and quality for rice fields. This study reports the complete chloroplast genome of *A. sinicus.* The chloroplast genome contained 110 complete genes, including 76 protein-coding genes, 4 ribosomal RNA genes, and 30 tRNA genes with 123,830 bp in length and a 34.66% GC content with IR loss. The evolutionary history, referred to as the maximum-likelihood (ML), showed that *A. sinicus* and *Astragalus bhotanensis* were most closely related. The chloroplast genome analysis of *A. sinicus* will serve as a reference for future studies on species evolution, plant conservation, and molecular phylogeny in *Astragalus*.

## Introduction

*Astragalus sinicus* Linne 1767, also known as Chinese Milk Vetch, is native to China and nowadays grows throughout southern China, Japan, and Korea (Li et al. 2008). Due to its nitrogen fixation capacity through symbiotic modulation with rhizobia, *A. sinicus* has become valuable green manure in organic farming and crop rotation (Chang et al. 2022). *Astragalus sinicus* promotes rice straw decomposition and decreases nitrogen runoff in rice-growing regions (Qiao et al. 2021; Zhou et al. 2021). Long-term application of *A. sinicus* in paddy significantly alleviates the bacterial community structure disorders caused by nitrogen fertilizer overuse, thereby improving rice’s growth and development habitat (Li et al. 2008; Zhang et al. 2017; Ma et al. 2018). To the best of our knowledge, research regarding *A. sinicus* is comparatively limited, and its chloroplast genome has been reported. This study *de novo* assembled the complete chloroplast genome of *A. sinicus* and investigated its phylogenetic relationships with other related species, providing a valuable resource for further research on species evolution, plant conservation, and molecular phylogeny.

Samples from Jiangxi Agriculture University (Qingshanhu, Nanchang, China; coordinates: 28.7597 N, 115.8352 E) were collected for sequencing. A specimen was deposited at the College of Bioscience and Engineering, Jiangxi Agricultural University (http://shenggong.jxau.edu.cn/, Licao Cui, email: cuilicao@jxau.edu.cn) under the voucher number KQnum20210807003. Genomic DNA was extracted from fresh seedling leaves using the modified CTAB method (Doyle and Doyle 1987). A library with an insert size of 400 bp was constructed. the chloroplast genome was sequenced with 150 bp paired-end reads using the Illumina NovaSeq 6000 system (Personalgene, Nanjing, China). After removal of adaptor contamination and low-quality reads, a total of 4,687,222 clean reads were obtained for the entire DNA. The A5-MiSeq v20150522 (Coil et al. 2015) and SPAdes v3.9.0 (Bankevich et al. 2012) were used to *de novo* assemble the chloroplast genome with default parameters. The assembled sequences were orientated using the BLAST v2.12.0 (Boratyn et al. 2013) and MUMmer v3.1 (Kurtz et al. 2004) programs with *Astragalus bhotanensis* (NC_047381.1) as a reference sequence. Gene annotation was performed using CPGAVAS v2 (Shi et al. 2019).

The complete chloroplast genome of *A. sinicus* (GenBank accession number: OM287552) remains a cyclic form with IR loss, consistent with other *Fabaceae* species (Liu et al. 2020; Ding et al. 2021; Guo et al. 2021). The chloroplast genome encoded 110 genes, including 76 protein-coding genes, 4 rRNA genes, and 30 tRNA genes. The total length was 123,830 bp and the overall GC content was 34.66%.

**Figure 1.**
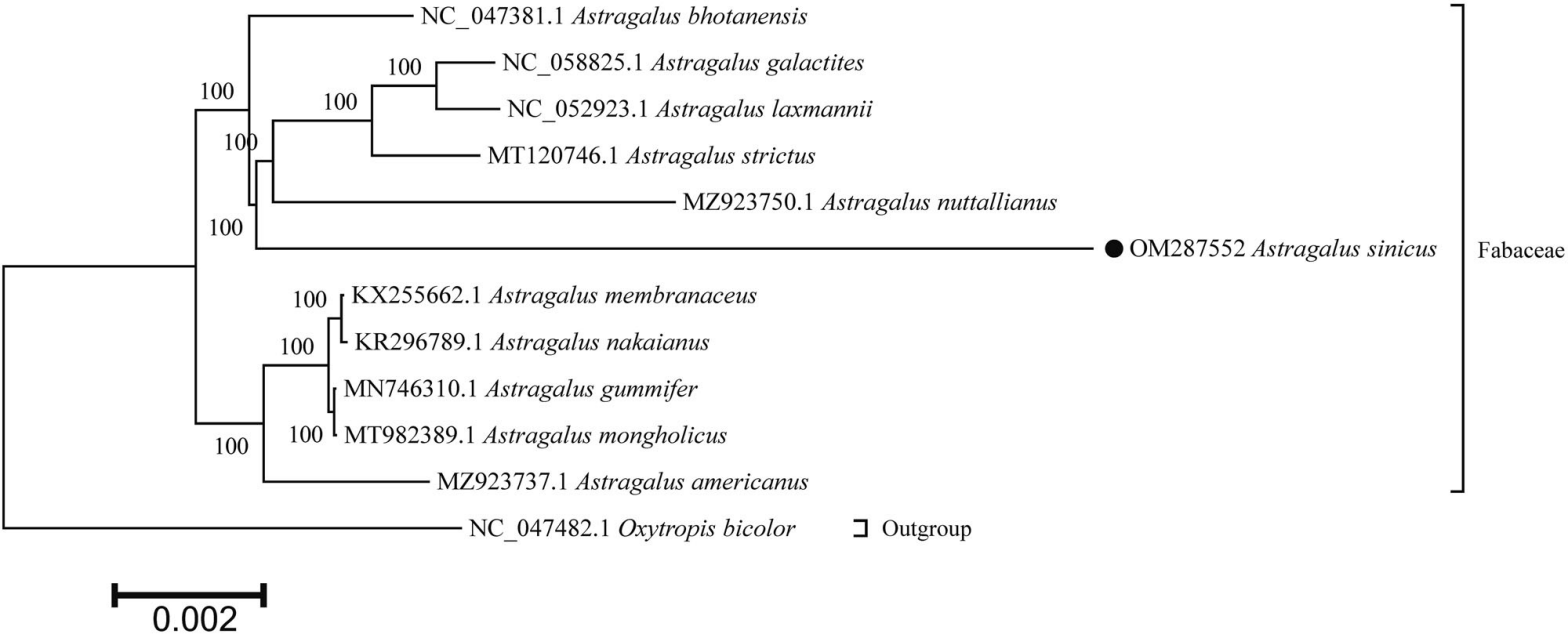
ML phylogenetic tree was constructed using RAxML v.8.2.11 based on the whole chloroplast genome sequences of 12 species. Numbers on branches are bootstrap support values from 1000 replicates.

To further investigate the *A. sinicus* phylogenetic position, the chloroplast genome sequences of 10 *Astragalus* species and *Oxytropis bicoloras* were downloaded from NCBI to construct the phylogenetic tree, with *O. bicoloras* being an outgroup. Multiple sequence alignment of the full-length chloroplast genome sequences was performed using MAFFT v.7.313 (Katoh and Standley 2013). Then, a maximum-likelihood (ML) phylogenetic tree was constructed by RAxML v.8.2.11 (Stamatakis 2014) under the GTRCAT model with 1000 bootstrap replicates (Figure 1). The phylogenetic tree showed that *A. sinicus* clustered with *A. bhotanensis*, *Astragalus galactites*, *Astragalus laxmannii*, *Astragalus strictus*, and *Astragalus nuttallianus* with high support values, in line with previous reports (Ding et al. 2021). The *A. sinicus* displayed the closest phylogenetic relationships with *A. bhotanensis*. The *A. sinicus* complete chloroplast genome provides useful information for the taxonomy, evolution, and molecular biology study of genus *Astragalus*, and might also facilitate the genetic research and breeding of *A. sinicus*.

## Data Availability

The data that support the findings of this study are openly available in GenBank of NCBI at https://www.ncbi.nlm.nih.gov, reference number OM287552. The associated BioProject, SRA, and Bio-Sample numbers are PRJNA802723, SRR17857198, and SAMN25582331, respectively.
